# Parental Perception of Childhood Anaemia and Efficiency of Instrument Assisted Pallor Detection among Mothers in Southeast Nigeria: A Field Validation Study

**DOI:** 10.1155/2019/7242607

**Published:** 2019-08-19

**Authors:** Maduka Donatus Ughasoro, Anazoeze Jude Madu, Iheoma Clara Kela-Eke, Uzoamka Akubuilo

**Affiliations:** ^1^Department of Paediatrics, University of Nigeria, Enugu Campus, Enugu, Nigeria; ^2^Department of Haematology, University of Nigeria, Enugu Campus, Enugu, Nigeria; ^3^Department of Haematology, University of Nigeria Teaching Hospital, Ituku/Ozalla, Enugu, Nigeria; ^4^Department of Paediatrics, University of Nigeria Teaching Hospital, Ituku/Ozalla, Enugu, Nigeria

## Abstract

**Background:**

Control of anemia can be achieved with early detection of pallor by parents at home. However, most parents lack the capacity to recognize pallor; thus most cases of anaemia are detected during hospital visit due to other symptoms. This study aimed to evaluate parental ability to detect pallor when aided with the anaemia screening tool.

**Methods:**

In the study information on the symptoms of illness and parental knowledge on anaemia. Their ability to detect anaemia aided with the Home-Base anaemia-screen tool (HB-Anae) was compared to the healthcare providers' assessment of pallor. The haemoglobin estimation with the Hb-301 haemoglobinometer was used as the gold standard.

**Results:**

None of the children in their previous illnesses had paleness as a complaint. Few (20.8%) parents knew what anaemia meant. Only 18.3% knew sites on the body where pallor can be detected. Many (55.1%; 304/552) surveyed children were anaemic (Hb<11g/dl) based on HB 301. Majority (88.8%; 270/304) of the parents aided with the HB-Anae were able to detect pallor on the children who were anaemic compared to 95.1% (289/304) detected by healthcare workers unaided, and the difference was not statistically significant (p=0.25).

**Conclusion:**

There was poor knowledge on anaemia among parents. The ability of parents to detect anaemia could be improved with the simple HB-Anae screen tool.

## 1. Introduction

About 1.62 billion people are affected by anaemia, which is equivalent to 24.8% of the world population [1]. The preschool-age children have the highest prevalence [1, 2] and the low and middle income countries have the greatest number of affected individuals [2]. Anaemia has multiple causes: chronic blood loss, nutrient deficiencies, haemolytic disease conditions, haemoglobinopathies, and suppressed erythropoiesis [3–5]. Early detection and treatment of anaemia can halt progression to severe anaemia and risks associated with transfusion.

The prevalence of childhood anaemia is high as reported by both hospital-based and community-based studies [6–8]. Despite this existing high prevalence of anaemia among children, most parents recognize weakness as the main complaint among anaemic children well rather than paleness [9–11]. Improvement in the parental ability to recognize pallor will be a step towards controlling childhood anaemia. A way to achieve that is bridging the gap between presence of pallor and ability of parents to recognize it at home and present their children to the hospital due to paleness, rather than being only hospital-based findings during physical examination for other presenting symptoms [12, 13]. There are different point-of-care diagnostic tests for anaemia available [14–19] but deployment of such in individual homes was not feasible especially in low and middle income countries. The feasibility of detecting childhood anaemia at the household level is not currently realistic. The only feasible strategy is reliance on clinical detection of pallor at certain anatomical sites [20, 21] which has been found to correlate positivity with the presence of anaemia [22–24]. The expertise in the clinical examination is common with physicians and nonphysician healthcare workers, rarely among parents without any form of training in healthcare. An effort to improve parental ability to recognize pallor led to the development of a simple Home-Based Anaemia (HB-Anae) Screening tool.

In this study, we evaluated the parental perception of anaemia and their health-seeking behaviours. We also evaluated the ability of parents to recognize pallor using the HB-Anae compared to both the haemoglobin level of less than 11 g/dL obtained using HemoCue 301 and visually observed clinical pallor as observed by healthcare workers. The aim was to determine the discriminatory ability of this screening tool in detecting childhood anaemia when applied by the parents on their children in the community as well as determine the sensitivity and specificity of the anaemia screening tool, compared with point-of-care HemoCue Haemoglobinometer. The finding would be relevant in the revision of the algorithm for the management of childhood anaemia.

## 2. Methods

The study was conducted in two urban and two rural communities in two states, in southeast Nigeria, namely, Abakpa (urban) and Ibagwa (rural) in Enugu East Local Government Area (LGA) of Enugu State as well as Umuahia (urban) and Nkwegwu (rural) in Umuahia North LGA, in Abia State. The study took place in July 2018. Households with children under 10 years of age were identified and their consent sought to allow their children to participate in the study. The mothers of these children were educated on how to use the HB-Anae.

### 2.1. Patient Recruitment

One local government area (LGA) was randomly selected from the list of LGAs in each of the study states. In the selected LGA, one rural ward and one urban ward were randomly selected. The wards were grouped in clusters according to geographical locations and one cluster was selected from each ward. A cluster comprises streets in the urban area and hermits in the rural areas. Individual houses of households with children under 10 years were identified and numbered. The households were systematically selected in an alternate of two, and selected households were informed about the study and those that showed willingness to participate were invited to the recruitment centers (health centers).

### 2.2. Data and Sample Collection

Interviewer-administered questionnaire was used to collect information on the child's previous medical history, caregivers' knowledge of anaemia, their knowledge on the causes of anaemia, how anaemia can be detected, and their health seeking behavior if their child is anaemic. The meaning of anaemia was explained to caregivers who did not know what anaemia means.

The HB-Anae was then explained to the mothers, who were also taught how to use the device. The HB-Anae screen tool is a device for detecting childhood anaemia. The tool is a double colour shaded device, which does not require any form of venipuncture or pin-prick bloodletting. It is designed to be used to compare for the reddishness and paleness of the pallor detecting sites: conjunctiva, palm, and nail bed. The kit requires minimal training and is reusable on multiple children. It does not require any form of consumables: cuvettes, stripes, reagent, and power. It is not temperature sensitive and can be stored at room temperature on a dry surface. The mothers were informed that the lighter shade of the screen represents low blood level and the darker shade represents normal blood level. They were taught to compare the shades with the palm, hue of the conjunctivae, and the nail bed. The mothers' ability to use the device was ascertained before they began to use it on their children by trying it on a palm of a normal child used as a simulator. An anaemic palm was created by momentarily occluding arterial blood supply to the palm into a fist and gently opening the palm. Previous pictures of anaemic children were also shown to the mothers to ensure they appreciate anaemia and not anaemia. See Supplementary Figures s1–s3. This was to ascertain that the mothers have learnt how to use the device before trying it on their children. Therefore, they were asked to use the device to categorize whether their children were anaemic or not, based on their observation at the three common sites: conjunctiva, palm, and nail bed.

A researcher observed the mothers check for pallor on their children using the HB-Anae and documented their assessment. Two independent healthcare providers also blindly assessed clinically the presence or otherwise of pallor, not using the anaemia screen. Conjunctivae are pale when the lower palpebral is pale. Gentle pressure was applied on the thenar eminence of the palm to check for paleness. The crease was avoided because in most dark skinned people the palmar creases are pigmented. The nailbed colour was assessed for paleness. Their findings are documented differently and immediately reviewed by a third clinician. In anywhere findings differed, a third clinician reviewed the person and the finding settles whether the child will be classified as pallor or not. A haemoglobin estimation was done by a phlebotomist who did venepuncture to collected blood in the cuvette of the HemoCue 301 machine. All the procedures of blood collection adhered to the World Health Organization (WHO) standard of universal precautions [25]. Anaemia children were given haematinic and referred to pediatric clinic for further evaluation.

Healthcare providers assessed clinically the level of pallor and categorized the same child whether anaemic or not.

### 2.3. Data Analysis

The data was entered in SPSS version 20. Parents and the healthcare workers categorized the children as either anaemic or not, based on detection of pallor at any of these sites: conjunctiva, palm, and nail bed. The children were grouped as not anaemic based on WHO cut-off of ≥ 11 g/dl and anaemic if less than 11 g/dl [26]. In this study, sensitivity and specificity of HB-Anae were based on comparing the outcome of the HB-Anae with the HemoCue HB results. Sensitivity is true positive/true positive plus false negative, and specificity is true negative/true negative plus false positive. True positives were those children categorized as anaemic by HB-Anae that were found to be anaemic according to the HemoCue 301. True negatives were those children categorized as nonanaemic by the HB-Anae that were found to be nonanaemic according to the HemoCue 301. False positives were those children categorized as anaemic by the HB-Anae that were found to be nonanaemic according to the HemoCue 301. False negatives were those children categorized as nonanaemic by the HB-Anae that were found to be anaemic according to the HemoCue 301. The significance was p value of ≤0.05.

The parents' responses to the question whether they knew what the word “anaemia” meant were categorized into “yes” or “no.” Those who claimed to know what it meant were asked to explain it, and those who did not know were given explanation. They were also asked to identify the sites where anaemia can be detected. They were further asked to state the action they will take if their child was found to be anaemic.

The respondents were asked to grade the fatality associated with childhood anaemia, using Likert-type scale which ranged as “not at all deadly,” “not too deadly,” “somewhat deadly,” “deadly,” and “very deadly.” Strong agreement “very deadly” was given the score of “5,” and it indicates that anaemia is very likely to cause death, and strong disagreement “not at all deadly” was given the score of “1” and a low probability that anaemia can cause death. The order of the scale is as follows: “not at all deadly” = 1, “not too deadly” = 2, “somewhat deadly” = 3, “deadly” = 4, and “very deadly” = 5. The illness history of their children within the preceding six-month period was based on parental call.

## 3. Results

Out of 573 children who were involved in the study, 21 were noted as invalid due to incompletely filled questionnaires and refusal to undergo further pin-prick after initial failed attempt, leaving 552 who were evaluated with point-of-care HemoCue 301 haemoglobinometer. Some 289 households were surveyed to obtain the 552 children involved in this study. Of the 552 children under 10 years recruited in this study, 176 (31.9%) and 112 (20.3%) were tested at Abakpa (Enugu, urban) and Ibagwa (Enugu, rural), respectively, and 148 (26.8%) and 116 (21%) were tested at Umuahia North (Umuahia, urban) and Nkwegwu (Umuahia, rural). Their ages ranged from 1 month to 120 months (mean 46.65 ± 34.2). The proportion of the children that were male and female were 313 (56.7%) and 239 (43.3%), respectively.

Among these children, 416 (87.8%) had sought for healthcare within the preceding 6 months (Table 1). Their health-seeking behaviours were as follows: patent medicine vendor/chemist (210/412, 51%), public hospital (104/412, 25.2%), private hospital (61/412, 14.8%), self-medication (31/412, 7.5%), and traditional/spiritual intervention (6/412, 1.5%). Among their reported symptoms were fever (76/78, 97.4%), cough (36/78, 46.2%), diarrhea (6/78, 7.7%), vomiting (4/78, 5.3%), seizure (4/78, 5.3%), and nasal discharge (3/78, 3.8%).

Out of the 289 respondents, 60 (60/289, 20.8%) knew what anaemia was. After explaining anaemia to the respondents, 34.3% (101/295) did not know how to detect it, 21.7% (64/295) would do a laboratory test, 20.3% (60/295) said it can be detected from skin colour, 15.6% (46/295) checked the eyes, and 8.1% (24/295) checked the palm and finger. Their intended actions if their child is found to be anaemic were as follows: blood tonic (130/275, 47.3%), vegetable (106/275, 38.5%), taking to hospital (94/275, 34.2%), feeding (56/275, 20.4%), drugs (32/275, 11.6%), transfusion (9/275, 3.3%), and antimalarial (7/275, 2.5%).

**Figure 1:**
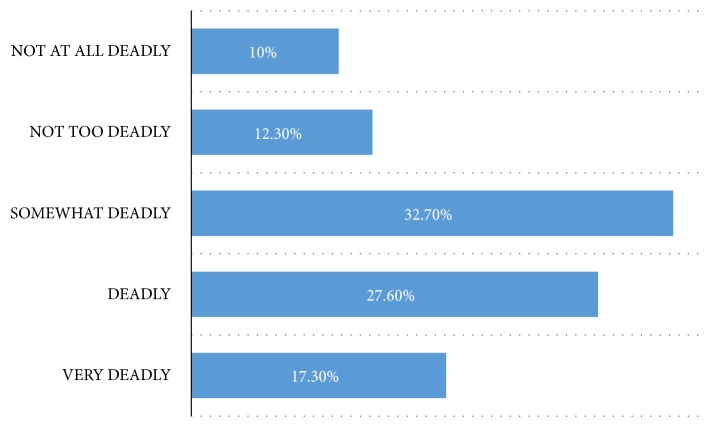
The rating of mortality of childhood anaemia by parents/caregivers.

32.5% (86/265) and 28.3% (75/265) of the respondents rated anaemia to be “somewhat deadly” and deadly, respectively (Figure 1). 17.4% (46/265) rated anaemia as very deadly.

About 270 (270/552, 48.9%) children were found to be anaemic by parents clinically using HB-Anae, compared to 289 (289/552, 52.4%) children found to be anaemic by clinical assessment by healthcare workers unaided, and the difference was not statistically significant (p = 0.25). The sensitivity, specificity, and positive predictive and negative predictive value of the HB-Anae when used by caregivers/parents and the ability of healthcare workers to detect anaemia unaided, both compared to the Hemocue 301 as the standard, were represented in Table 2. The HB-Anae had sensitivity of 58.9% and specificity of 63.3%, while healthcare workers detection of anaemia clinically had sensitivity of 68.1% and specificity of 62.9% (Table 2). The positive predictive value (PPV) and negative predictive value (NPV) of the HB-Anae were 66.3% and 55.7%, respectively, while the PPV and NPV of unaided clinical detection of anaemia were 71.6% and 62.4%, respectively.

**Figure 2:**
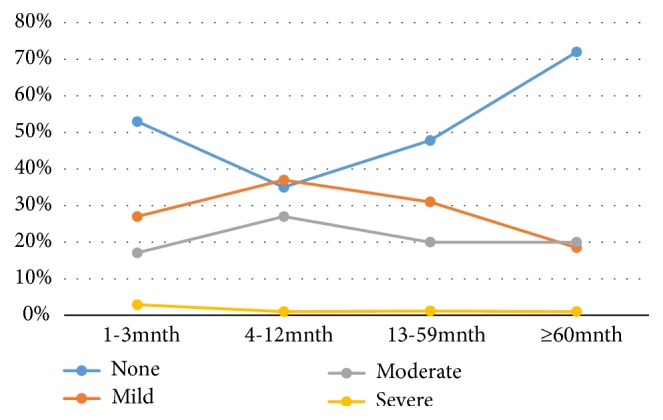
Prevalence of anaemia in children.

The higher (64.3%) proportion of children within the age range of 4–12 months had anaemia, followed by 51% of those within the age range of 13 to 59 months. The least 28.6% were within the age range of 60 months and above. The proportion of children within the age range of 4-12 months with mild and moderate anaemia were 38% and 27%, respectively (Figure 2).

## 4. Discussion

The prevalence of childhood anaemia was high and is similar to what has been reported [6, 27]. According to the WHO classification of anaemia in a population based on the prevalence estimated from blood levels of haemoglobin, it has exceeded the 40% cut-off for severe category [2, 28]. When compared to the previous WHO estimates [29], it indicates that Nigeria has not made much impact on its fight to control anaemia, at least in the region where the study was conducted. Thus, there is need to reevaluate the existing anaemia control strategies in Nigeria, to ensure that determinants of childhood anaemia are identified and adequately addressed. However, while effort is being made to identify how best to improve on the Nigeria anaemia indices, resources should be channeled to improved utilization of healthcare facilities by those children who were found to be anaemic. Our findings confirm that most parents utilized the services of patent medicine vendors when their children were ill. This finding was consistent with findings in other published studies of health-seeking behavior [30–33]. It is conflicting to note that the majority accepted to take their children to hospital if found to be anemic but in the previous healthcare they sought, they utilized services of patent medicine vendors and self-medication, which is similar to what has been reported [32, 33]. This discrepancy may not easily be explained. But none of the parents reported paleness as one of the symptoms of their children's previous illness. It is possible that when parents start to recognize pallor, their attitude might change since most parents lack the ability to detect anaemia and categorized anaemia as deadly as revealed in this study, as well as the established relationship that exists between childhood illnesses and anaemia [22–24].

Our experience demonstrated that, with simple aid, parents and caregivers (nonphysician; non-healthcare workers) can detect pallor in their children. The HB-Anae performed reasonably well to detect childhood anaemia at the three cardinal sites when implemented by parents and caregivers, who received short training. Their detection of anaemia compared reasonably well with the clinical assessment for pallor done by healthcare workers. The sensitivity and specificity of HB-Anae by parents and clinical pallor by healthcare workers were high at haemoglobin concentration of 11 g/dl. This is similar to what Stoltzfus et al. [34] reported for clinical assessment of pallor. When the HB-Anae was compared with clinical assessment by healthcare workers, both the sensitivity and the specificity were increased. The high prevalence of childhood anaemia reported in this study shifts emphasis to the positive predictive value of the HB-Anae, meaning that if a child is found to be anaemic, he/she is more likely to be found to be anaemic by laboratory evaluation. This made this finding an important one and can be a good assessment tool for childhood anaemia in African homes where prevalence of anaemia is high. This is a screening tool which is meant to be applied among asymptomatic individuals who may or not have anaemia, instead of a diagnostic test tool which is used to determine the presence or absence of anaemia when the subject has shown signs of anaemia: weakness and dizziness. Applying this tool ahead of a diagnostic test on the HemoCue or WHO Haemoglobin colour scale will increase early detection of children anaemia.

It is striking to note that majority of the respondents did not know when a child is anaemic. This underscores that finding in this study, where paleness was not among the complaints of the children that utilized hospital facilities recently. Therefore there is a need to raise their awareness about anaemia as well as how to detect it in their children.

Some limitations exist in the study, namely, the omission of testing for the ability of parents to detect anaemia unaided, so as to have a reference to compare the HB-Anae outcome. This omission might have led to overestimation of the true impact of the HB-Anae. Since, conventionally, most examiners for clinical pallor often use their own palm to check for pallor rather than patients' palms and nail bed, having a standard reference like the HB-Anae will reduce the effect if the parents are pale themselves. Furthermore, the design of the study did not make provision for validation of the HB-Anae at different hemoglobin levels, as well as different childhood age groups. This will be another research area, since it will add external validity to the HB-Anae. Another limitation was the inclusion of infants in the study. Hemoglobin limits that define anemia differ to ages, with infants having a relatively higher value of haemoglobin concentration (12g/dl) as anaemia compared to children 2–10 years (11 g/dl). It would be more appropriate to limit patients included in this study to children between 2 and 10 years of age. However, what this study evaluated was ability of caregivers to clinically detect pallor in their children aided or unaided, not necessarily their haemoglobin levels, and the ability of HB-Anae to detect moderate to severe anaemia even among infants still remains undeterred.

## 5. Conclusion

The study yielded that when the HB-Anae was used by parents and caregivers to detect pallor on their children, it compared favorably with clinical detection of pallor by healthcare workers, but poorly with laboratory detection of anaemia. This makes it a good screening tool but an imperfect diagnostic tool which can bring the detection of pallor in the children's closet caring environment (home). Though treatment for anaemia cannot be based on the outcome of use of the HB-Anae, it can be incorporated into childhood anaemia control algorithm in regions with high prevalence of anaemia to expand the pool of apparently ill children, the proportion that are likely to visit hospital for care rather than conventional patent medicine vendors or resort to self-medication. If deployed, the HB-Anae could serve as regular reminders to parents and caregivers on what normal nonanaemic mucosae look like in order to be equipped to distinguish pallor from non-pallor.

## Figures and Tables

**Table 1:** Parental knowledge of anaemia and children's previous medical history and their health-seeking behavior.

Variables	n	%
Was the child sick within the previous 6 months (n=474)		
Yes	416	87.8
No	58	12.2
The symptoms children previously presented with when ill (n=78)		
Fever	76	97.4
Cough	36	46.2
Diarrhea	6	7.7
Vomiting	4	5.3
Seizure	4	5.3
Nasa discharge	3	3.8
Health-seeking behavior (n=412)		
Patent medicine vendor	210	51.0
Public hospital	104	25.2
Private hospital	61	14.8
Self-medication	31	7.5
Traditional/Spiritual	6	1.5
Parents' knowledge of anaemia (n=289)		
Yes	60	20.8
No	229	79.2
How to detect pallor (n=295)		
Don't know	101	34.3
Test	64	21.7
Skin color	60	20.3
Paleness	54	18.3
Eyes	46	15.6
Hands & Feet	24	8.1
Action to take if a child is found to be pale (n=275)		
Blood tonic	130	47.3
Give vegetable fruits	106	38.5
Take to Hospital	94	34.2
Adequate feeding	56	20.4
Drugs	39	14.2
Transfusion	9	3.3

**Table 2:** The distribution of clinical pallor and low haemoglobin in the four study localities and sensitivity, specificity, and positive and negative predictive value of HB-Anae in detecting anaemia compared to HemoCue.

Locality (n)	Proportion with pallor detected at any site by parents using HB-Anae	Proportion with pallor detected at any site by healthcare workers	Proportion with low haemoglobin using HemoCue 301 (<11g/dl)	p-value
	n (%)	n (%)	n (%)	
Prevalence of anaemia (552)	270 (48.9)	289 (52.4)	304 (55.1)	
Enugu				
Abakpa (176)	63 (35.8)	48 (27.3)	59 (33.5)	0.25
Ibagwa (112)	45 (40.2)	66 (58.9)	77 (68.8)	(1.303)
Umuahia				
Umuahia North (148)	92 (62.2)	96 (64.9)	96 (64.9)	
Nkwegwu (116)	70 (60.3)	79 (68.1)	72 (62.1)	
HemoCue as Standard				
Sensitivity	179/304 (58.9%)	207/304 (68.1%)		
Specificity	157/248 (63.3%)	156/248 (62.9%)		
Positive Predictive Value	179/270 (66.3%)	207/289 (71.6%)		
Negative Predictive Value	157/282 (55.7%)	156/263 (62.4%)		
HB-Anae compared with Clinical assessment by HCWs				
Sensitivity	221/287 (77.0%)			
Specificity	199/235 (84.7%)			
Positive Predictive Value	221/257 (86.0%)			
Negative Predictive Value	199/265 (75.1%)			

## Data Availability

The data used to support the findings of this study are available from the corresponding author upon request.

## Abbreviations

HB: Haemoglobin
LGA: Local Government Area
WHO: World Health Organization.

## Additional Points

*Learning Points. What is already known on this topic?* Anaemia is common among children. Most mothers/caregivers lack the ability to notice when a child is anaemic. Most cases of anaemia are detected only during routine facility-based physical examination of their children.* What does this paper add*? Most mothers/caregivers with proper training aided with the Home-Base anaemia-screen can recognize when a child is pale. Their ability to detect pallor compares favorably with that done by physicians when equipped with Home-Base anaemia-screen. Most of the mothers/caregivers were willing to bring their children to a health facility if found to be pale.

## Supplementary Materials

Supplementary Materials Figure S1: palm of a child with severe pallor. Figure S2: sole of foot of a child with moderate pallor. Figure S3: sole of the foot of a normal child without pallor.
