# Supplementary material for: Parental Perception of Childhood Anaemia and Efficiency of Instrument Assisted Pallor Detection among Mothers in Southeast Nigeria: A Field Validation Study
Source: Int J Pediatr. 2019 Aug 19;2019:7242607. doi: 10.1155/2019/7242607 (PMC6719268; doi:10.1155/2019/7242607)
Supplement: Supplementary Materials — Figure S1: palm of a child with severe pallor. Figure S2: sole of foot of a child with moderate pallor. Figure S3: sole of the foot of a normal child without pallor. [file 7242607.f1.doc]

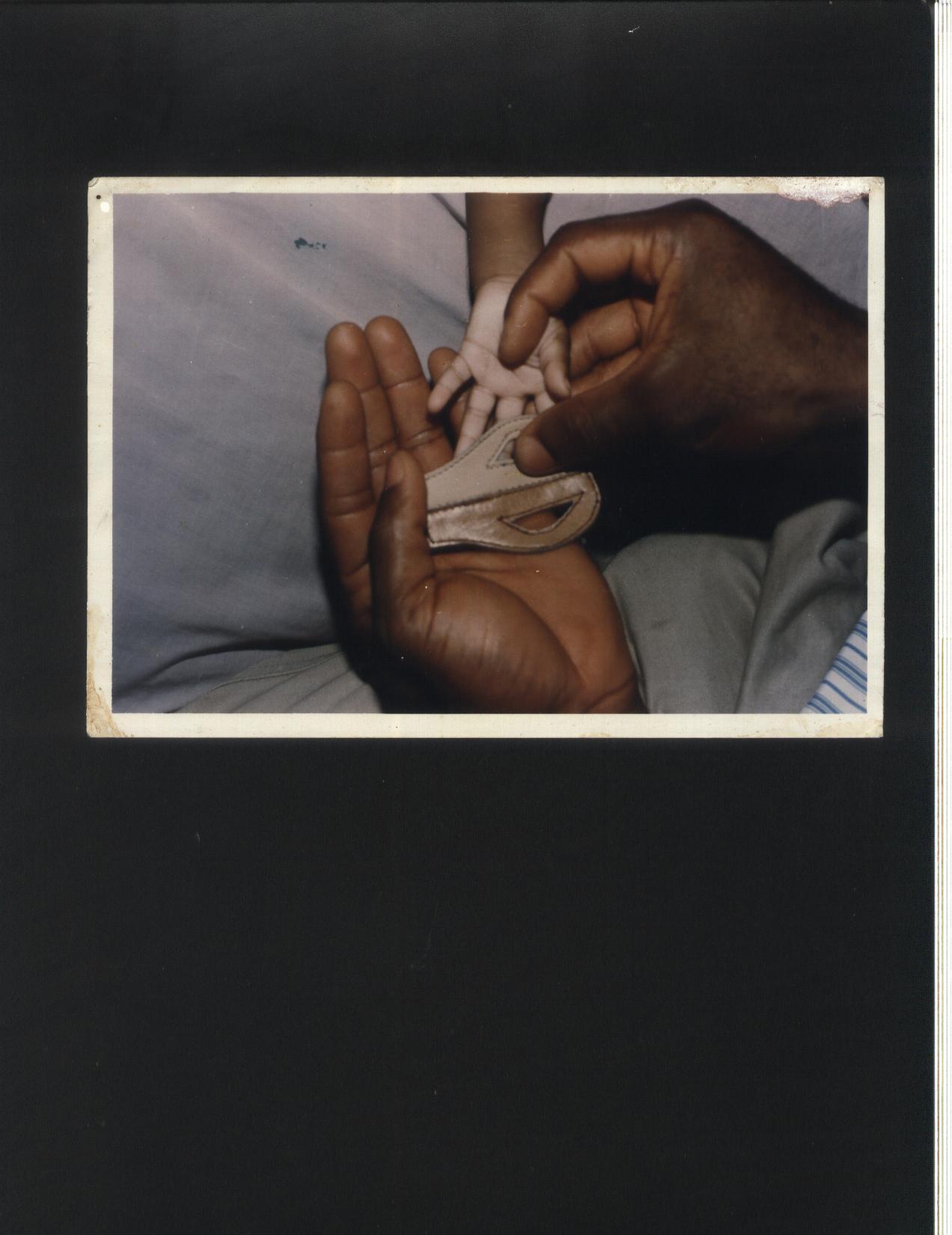


Figure S1. Palm of a child with severe pallor


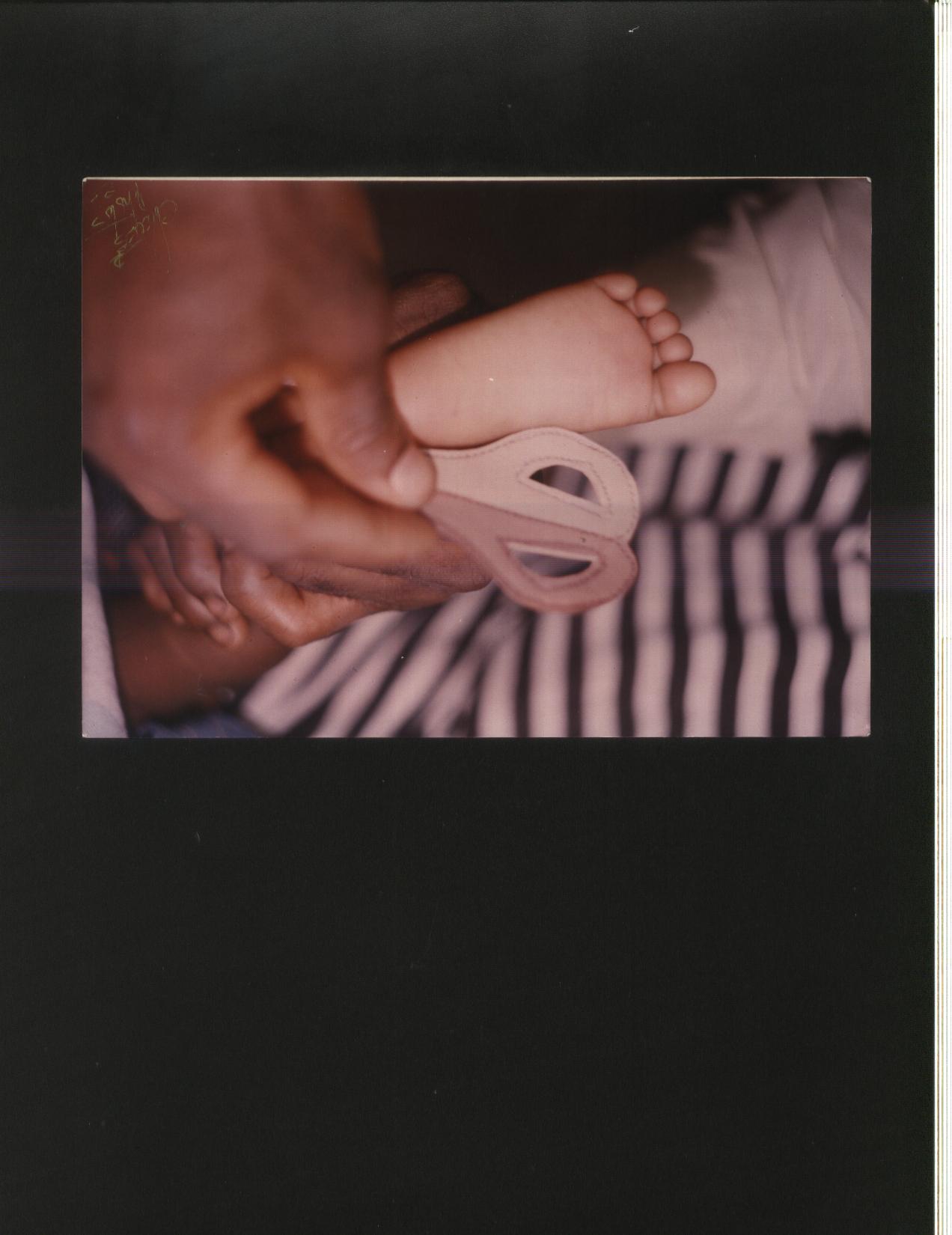


Figure S2 Sole of foot of a child with moderate pallor


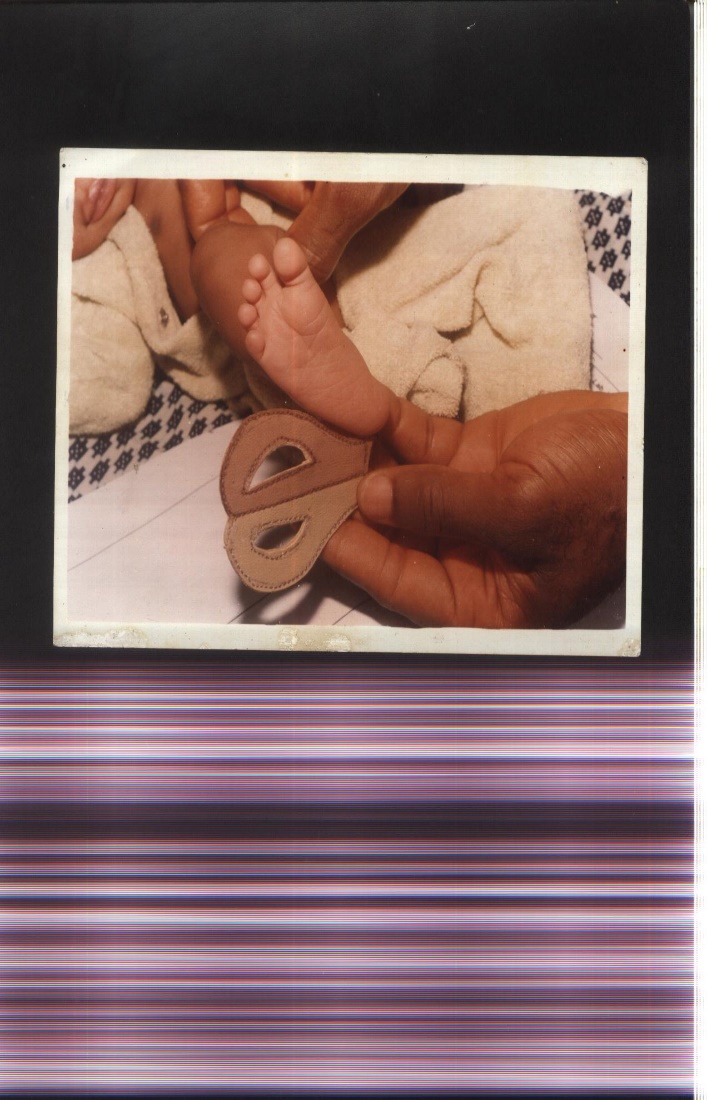


Figure S3. Sole of the foot of a normal child without pallor.
